# Biomechanical investigation of elbow dislocation: comparative analysis using *Papio anubis* baboon and human cadaver models

**DOI:** 10.3389/fbioe.2025.1630615

**Published:** 2026-03-19

**Authors:** Samer Al Kork, Karim Youssef, Sherif Said, Taha Beyrouthy, Farid Amirouche, Edward Abraham

**Affiliations:** 1 College of Engineering and Technology, American University of the Middle East, Egaila, Kuwait; 2 Department of Orthopaedic Surgery, University of Illinois at Chicago, Chicago, IL, United States

**Keywords:** biomechanics, elbow dislocation, fracture, collateral ligaments, *Papio anubis* baboon, human cadaver, sports injury, stages of dislocation

## Abstract

This study investigates the biomechanical mechanisms underlying elbow dislocation, emphasizing the role of flexion angle and forearm rotation on joint stability. Simulating realistic fall dynamics and injury conditions remains a major challenge in experimental biomechanics, and this work addresses that gap through controlled *in vitro* testing and computational modeling. Seventy *Papio anubis* (baboon) and twenty-one human cadaveric arms were tested under axial and hyperextension loading conditions to evaluate dislocation thresholds and ligament failure sequences. These trials indicate that maintaining bone integrity and soft-tissue support may restore elbow stability through severalnonsurgical strategies. Across both models, dislocation resistance increased with elbow flexion and was significantly greater in pronation compared to supination. The results demonstrate that maintaining bony congruence and soft-tissue integrity substantially enhances stability and that complete dislocation typically requires combined ligament rupture and bony failure. Across 0°–45° of flexion, Stage III dislocation thresholds reached approximately 1.9–2.2 kN in pronation versus 0.8–1.0 kN in supination for *Papio anubis*, closely matching the human mean of 1.94 kN. Finite-element simulations confirmed these patterns, revealing stress localization at the coronoid process and radial head consistent with early-stage dislocation. The results highlight the translational relevance of the baboon model for studying human elbow instability and provide a validated framework for future surgical and rehabilitation strategies. These findings advance the mechanical understanding of elbow instability and emphasize how forearm orientation and flexion angle influence load distribution, ligament strain, and the sequence of failure.

## 1 Introduction

The elbow is the second most frequently dislocated major joint in adults and the most frequently dislocated in young people (Kuhn and Ross, 2008; Barco et al., 2023; Hyvönen et al., 2019). Elbow dislocations range from simple to complex. Complex acute dislocations may involve fractures, patient apprehension, and pain (Barco et al., 2023) and may require surgical intervention (Cohen and Hastings, 1998). Simple dislocations are defined as those accompanied only by small avulsions (1–2 mm) or limited to soft-tissue injury (Josefsson et al., 1984; Robinson et al., 2017; Schnetzke et al., 2021). Some patients show no residual symptoms (Josefsson et al., 1984). Most simple dislocations can be managed nonoperatively with good long-term outcomes without surgical interventions (Robinson et al., 2017) by immobilization, while some do require surgeries (Mühlenfeld et al., 2022). Untreated posterior dislocation often leads to stiffness, pain, and deformity (Pal et al., 2021).

In sports and daily activity, the elbow frequently experiences complex loading that combines axial compression, valgus stress, and rotational torque. Forceful impacts—such as spiking in volleyball, throwing in baseball, or blocking a shot in soccer—can drive posterior dislocation by coupling hyperextension with forearm rotation. Injuries to the elbow, forearm, and wrist together account for roughly one-quarter of all sports-related upper limb injuries (Magra et al., 2007), though their frequency varies by sport and position. Elbow dislocation may also result from falls from height, trampoline use in children, or industrial accidents (Josefsson and Nilsson, 1986; Lewallen et al., 2023; Gong et al., 2023).

Table 1 summarizes most common elbow injury that occurs in most common played sports ^4^ as reported from (Magra et al., 2007).

**TABLE 1:** Sports and common elbow injuries (adapted from (Magra et al., 2007)).

Sport	Common injury
American Football	Valgus stress when throwing a pass; hyperextension and dislocation, and olecranon bursitis resulting from direct trauma
Baseball	Valgus stress of pitching: medial traction, lateral compression, posterior abutment
Basketball	Posterior compartment with follow-through on jump shot
Bowling	Flexor–pronator strain
Gymnastics	Radiocapitellar overload and posterior impingement during weight-bearing on the extended elbow
Soccer	Lateral epicondylitis through hyperextension of the elbow when blocking a shot
Volleyball	Valgus stress at impact of spiking
Weight lifting	Ulnar collateral ligament sprain, ulnar nerve irritation
Water skiing	Valgus extension overload of posterior compartment with trick skiing

**FIGURE 1:**
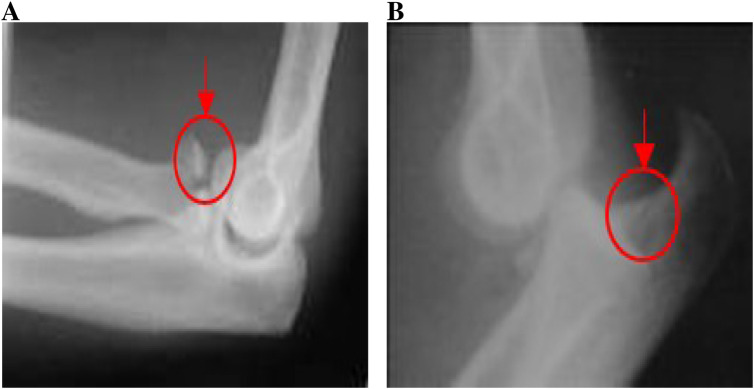
**(A)** Radial head fracture. **(B)** Coronoid process fracture.

Hildebrand et al., reported (Hildebrand et al., 1999) an annual incidence of approximately 6–8 cases per 100,000 people; these represent 11%–28% of all elbow injuries. In addition to dislocations at the elbow, there are different types of fractures that occur at the elbow from a number of these activities mentioned above. It’s also noted that 30% of elbow fractures in adults occur in the radial head. A fracture of the radial head (see Figure 1A). Olecranon process fractures account for 20% of all elbow injuries in adults. Coronoid process fractures occur in 10%–15% of dislocations of the elbow. Figure 1B illustrates a coronoid process fracture.

The mechanical pathway of elbow dislocation has been conceptualized through several landmark studies. O’Driscoll and colleagues (O’Driscoll et al., 1992; O’Driscoll et al., 2000) proposed a three-stage progression of posterolateral instability initiated by external rotation and valgus loading. Wake et al. (Wake et al., 2004) further demonstrated that axial compression at low flexion angles produces sequential fracture–dislocation patterns. Despite these advances, few studies have systematically compared the role of forearm rotation and flexion angle on dislocation thresholds under controlled loading.

To address this gap, the present work integrates two complementary experimental models—juvenile *Papio anubis* and human cadaveric elbows—to test the hypothesis that pronation and increased flexion enhance elbow stability by improving ulnohumeral congruence and ligament tension. Using standardized axial and hyperextension loading, the study quantifies dislocation thresholds, ligament rupture sequences, and fracture patterns across flexion angles. The comparative analysis between species establishes the translational validity of the baboon model and provides a biomechanical framework for understanding injury mechanisms relevant to sports and trauma surgery.

This paper aims to improve the understanding of elbow dislocation by exhibiting an experimental study on human cadavers and *Papio anubis* baboon arms in different scenarios. Indeed, as it will be shown in the paper, different aspects of similarity exist between baboon and human arms and allow to conduct cost-effective experiments on elbow dislocation with baboon arms. The study involves different arm configurations and loads with an observation of the sequences of events occurring in the different components of the arm and the elbow. The experimental investigation conducted with baboon and human cadaver arms allowed to construct a clear view of sequences of ligament ruptures and dislocation stages. As reported above, such an understanding is fundamental as it provides measures of prevention and treatment.

The paper is organized as follows. In Section 2, previous work on elbow dislocation mechanisms is presented. Section 3 presents the experimental procedure for baboon arms and human cadaver arms. Section 4 shows the experimental results obtained with both studies, followed by a discussion and a conclusion in Section 5.

## 2 Mechanics of elbow dislocation

The mechanism of elbow dislocation remains debated and has been described in multiple ways by different investigators (Robinson et al., 2017; Barco et al., 2023). It is essential to understand the factors that could lead to elbow injuries and instability and to establish measures of diagnosis and treatment (Mühlenfeld et al., 2022) or prevention in different cases, like pediatric sports in particular (Magra et al., 2007). Reconstructing falling is one of the most challenging problems in bio-mechanics. Current models which attempt to reconstruct falls usually focus on inverse dynamics where muscle forces are determined mathematically.

**FIGURE 2:**
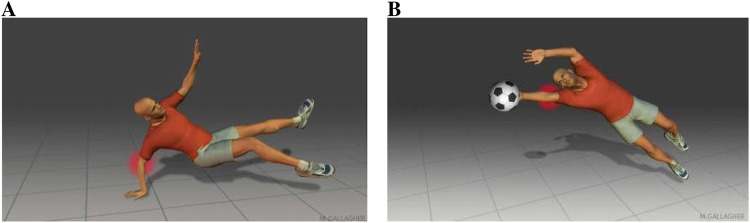
**(A)** Falling onto an outstretched hand. **(B)** Blocking a shot.

Different groups of instability and fracture dislocation patterns were shown in (Reichert et al., 2021) and were proposed to be helpful in recognizing the injury mechanism and possible treatments. These groups involve:

1. The terrible triad, an elbow dislocation affecting different parts of the elbow joint affected: fractured coronoid process fractured, disrupted soft tissues disrupted and others (Reichert et al., 2021; Kani and Chew, 2019).
2. The Monteggia fracture: a proximal third of the ulna fractured with the radial head dislocated (Reichert et al., 2021; Ring, 2013).
3. Groups for the fracture-dislocations of the following: posterior radial head, Anteromedial coronoid and Trans-olecranon. The most common mechanism for traumatic posterior elbow dislocations (accounting for approximately 80% of all dislocations (O’Driscoll et al., 2000)) typically occurs when an individual falls and lands on an outstretched hand, as illustrated in Figure 2A. Upon impact with the ground, this action exerts a compressive force on the elbow joint, leading to the dislocation. Typically, there is a turning motion in this compressive force. This can drive and rotate the elbow out of its socket. Elbow dislocations can also happen in car accidents when the passengers reach forward to cushion the impact. A decisive solid blow to the posterior aspect of a flexed elbow may result in anterior dislocation of the elbow. This force drives the olecranon forward in relation to the humerus. Anterior dislocations and any open fracture are commonly associated with disruption of the brachial artery and/or injury to the median nerve.

Hyperextension force at the elbow with forearm supinated is another known mechanism (Tyrdal and Olsen, 1998) illustrated in Figure 2B. Combined hyperextension and supination have been claimed as the cause of lateral epicondylitis if the pain is localized laterally. It was reported according to an epidemiological study that 75% of soccer goalkeepers experience elbow problems through their career in which 95% sustain pain through hyperextension of the elbow when blocking a shot (Tyrdal and Bahr, 1996).

### 2.1 Instability in elbow dislocation and subluxation

In an elbow subluxation and dislocation investigation (O’Driscoll et al., 2000; 1992), O’Driscoll et al., designed an experiment to test the validity of the hypothesis that the elbow can be dislocated posteriorly with a functionally intact anterior medial collateral ligament (AMCL) and also determine the mechanism or kinematics of such dislocation and its clinical relevance. Thirteen upper extremities (seven right, six left) were used in this study. The humerus was dis-articulated from the shoulder joint as well as the radius and the ulna was dis-articulated from the wrist. All non-ligament soft tissues were removed, keeping the tissue around the elbow joint and the tendon insertion of the biceps, brachialis and triceps intact. The humerus was securely fixed in non-magnetic plastic frame and positioned so that the forearm moved in a horizontal plane with flexion and extension of the elbow for optimal testing of valgus instability. Loads of 1 kg for the biceps and brachialis and 2 kg for the triceps were used to simulate muscle tone. A 3 Space Isotrak electromagnetic tracking system was fixed to the plastic frame to which the humerus is firmly secured. The sensor was attached to the proximal of the ulna. The origin and insertion of the AMCL was digitized. The distance between the humerus and the ulna was calculated in real-time. External rotation and valgus moment with axial forces resulted in posterior elbow dislocation in twelve of the thirteen specimens with anterior medial collateral ligament intact (AMCL). O’Driscoll et al., assumed that the mechanism of dislocation during a fall on the outstretched hand would involve the body rotating internally on the elbow (O’Driscoll et al., 2000; 1992; O’Driscoll, 1999), which experiences an external rotation and valgus moment as it flexes. Experimental results also showed that dislocation occurs at 80 degrees of flexion with poster lateral rotation of 34–50° and 5–23 degrees of valgus moment. He also suggested that posterior dislocation can be reduced in supination. O’Driscoll et al., defined dislocation as the final of 3 instability stages resulting from posterolateral rotation, with a disruption of soft tissues progressing from lateral side to medial side.

### 2.2 Elbow dislocation by axial compressive load

In another experimental and 2D Finite Element (FE) investigation shown in (Wake et al., 2004), Wake studied the bio-mechanical analysis of the mechanism of elbow dislocation by a compressive force.

Fifty-three intact elbows where chosen for this study, where the humerus was cut transversely 90 mm proximally to the distal joint surface. The radius and the ulna were transected evenly at a point either 60 mm (short ulna model) or 90 mm extended ulna model) distally to the Coronoid tip. In this study, the humerus, radius, and ulna were fixed in dental resin to a depth of 30 mm in the specimen holders of the loading apparatus. Axial compressive loads were applied at 10 mm/min while the elbow joint being flexed at 15° of extension and 0°, 30°, 60° or 90° of flexion in custom made apparatus.

The loading experiments produced various dislocations and fractures of the humeral shaft (13%), supracondyle (30%) and radial or ulnar shaft (28%). Anterior fracture-dislocation [type II: with Olecranon fracture at 60° flexion position or 90° flexion position occurred when the load was applied at 60° and 90° of flexion. In the 60° flexion position and 60 mm forearm length, type II or combined types I and II occurred.

Posterior fracture-dislocation [type I: with Coronoid fracture, at 15° extension, at 0° flexion position, and 30° flexion position occurred from 15° of extension to 30° of flexion. All cases had a concurrent coronoid process and radial head or neck fractures. At 90° of flexion, the humerus catches on the Olecranon to develop a fracture of the Olecranon, which is presumed to have caused the failure of the posterior supporting systems.

### 2.3 Elbow dislocation by hyperextension load

Hyperextension at the elbow joint is another known mechanism of elbow dislocation. Tydral designed in (Tyrdal and Olsen, 1998) an experiment to produce a combined hyperextension and supination at the elbow joint and observed the lateral ligament lesions induced. In this study, ten elbow cadavers from five male donors with a mean age of 28.8 years were used. Relatively young donors were chosen since age-related changes are commonly expected in human tissue and specimens near the same age range as active athletes were wanted. All skin and fatty tissues were dissected away leaving the ligaments around the elbow joint and the forearm muscles intact. A three-dimensional loading apparatus was developed to study the kinematics of elbow dislocation. The humerus was mounted horizontally in the loading apparatus, and fixed with one screw and four hose clamps. The rotation was blocked by one lateral screw.

The forearm was connected to the mobile lever arm by two screws through the proximal ulna. The nylon line was fixed to an eyelet screw going through the distal radius from the volar side. Hyperextension force was applied in the form of bags filled with an increasing amount of water. The bags were also allowed to fall from 1.5 to 2 m. The loads were applied at the elbow being in maximal extension and in full supination to imitate the kind of trauma cased by a handball. The impact of the falling bags corresponded to the effects of a handball (450 g) hitting the distal forearm at different speeds (Tyrdal and Bahr, 1996). The experimental loads applied at the last trauma corresponded to the speed of a handball between 65 and 200 km/h. The hyperextension loads resulted in three different injuries to the ligaments: (1) anterior capsule rupture, (2) avulsion of the proximal insertion of the medial and (3) the lateral collateral ligaments. The lesion was only visible from the inside of the joint, but in some cases the lateral lesion could also be seen from the outside. Cartilage lesions of the Olecranon or the humerus were not observed.

### 2.4 Posterolateral stability of the elbow

Another possible mechanism of elbow dislocation is recurrent posterolateral rotatory instability (Tyrdal and Bahr, 1996). The combination of elbow dislocation with fractures of the radial head and the coronoid process has been termed the terrible triad by Hotchkiss in (Hotchkiss, 1996). Schneeberger designed in (Schneeberger et al., 2004) an experimental setup to evaluate the role of the radial head and coronoid process as posterolateral rotatory stabilizers of the elbow joint. Ten fresh-frozen upper extremities cadavers with no evidence of pathological changes at the elbow were used for this study; two were used for a pilot evaluation, and eight were used for measurements. The limbs were amputated through the proximal third of the humerus and dis-articulated at the wrist. All soft tissue around the elbow joint was left intact during the study.

The upper arm was fixed to a specifically designed frame using a large AO external fixator with two bicortical 4.5-mm Steinmann pins placed through the humeral shaft. A standardized surgical approach was designed to gain access to the coronoid process and radial head for this experiment. The approach consisted of two osteotomies-one of the lateral epicondyle and one of the ulnar insertion of the lateral ulnar collateral ligament-performed with an oscillating saw. The posterolateral rotatory displacement of the ulna was measured after application of a valgus and supinating torque (1) in seven intact elbows, (2) after radial head excision, (3) after sequential resection of the coronoid process, (4) after subsequent insertion of each of two different types of metal radial head prostheses (a rigid implant and a bipolar implant with a floating cup, and (5) after subsequent reconstruction of the coronoid with each of two different techniques in the same Cadaver elbow.

This vivo study showed that the posterolateral rotatory laxity averaged 5.4° in the intact elbow at 60° of flexion. Excision of the radial head in an elbow with intact collateral ligament caused a mean posterolateral rotatory laxity of 18.6° (p < 0.0001). The elbows with a defect of 50% or 70% of the coronoid, loss of the radial head, and intact ligaments could not be stabilized by radial head replacement alone, but additional coronoid reconstruction restored stability. Resection of 30%, 50%, and 70% of the coronoid process resulted in detachment of the annular ligament at the base of the coronoid of approximately 50%, 70%, and 90%, respectively. In clinical replacement, replacing the radial head with a rigid implant seems to restore stability better than replacing a floating prosthesis.

### 2.5 The three-column concept

The three-column concept was proposed in (Watts et al., 2019) for elbow fracture with dislocations, into improving the understanding of injury patterns. The elements of the elbow joint were decomposed into three columns: medial, middle and lateral. This allowed for the proposal of a classification system for elbow fracture dislocations which can help in treating elbow injuries efficiently.

### 2.6 The “reversed Horii circle”

Magnetic resonance imaging and radiography datasets of 64 patients were used in (Schnetzke et al., 2021) to analyze the mechanism and injury patterns in elbow dislocations. Among the study’s findings, a “reversed Horii circle” was proposed, with a medial force of induction that originates and continues from medial to anterior. This term is used in the context of the “Horii circle”, the term used for the disruption of soft tissue from lateral to medial, described by O’Driscoll et al. (O’Driscoll et al., 1992; O’Driscoll, 1999)

## 3 Materials and methods

### 3.1 Experimental investigation of elbow dislocation in baboon arms

**FIGURE 3:**
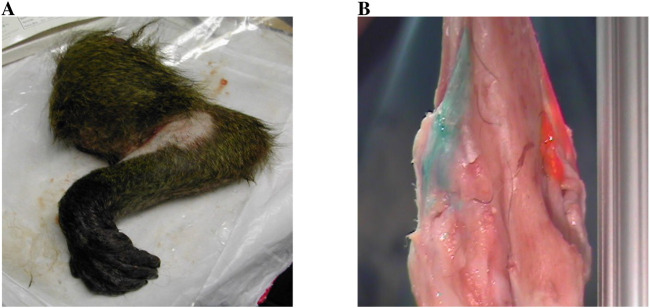
**(A)** One of the baboon arms used in the study. **(B)** Posterior View of AMCL (Pink), PMCL (Orange) and LCL (Green).

This study experimentally investigated elbow dislocation using the arms of the *Papio anubis* baboon, as illustrated in Figure 3A (Alkork, 2011). Baboons were selected for their close anatomical similarity to the human elbow joint and their accessibility through established university research programs. In addition, baboon arms provide a cost-effective and logistically feasible alternative to human cadavers, which are limited in availability, highly regulated, and exhibit greater variability in donor health and tissue quality. The smaller scale and consistent anatomical features of baboon limbs make them well suited for controlled experimental setups and reproducible mechanical testing. Collectively, these factors support the use of the baboon as a valid and ethical intermediary model for studying elbow dislocation mechanisms prior to translation to human studies. Seventy female *Papio anubis* (2–5 years; mean weight, 10.8 ± 1.3 kg) were divided into two main groups. Group I included 62 arms used for the mechanical analysis of elbow dislocation, and Group II included 8 arms reserved for anatomical dissection. The humerus was dis-articulated from the shoulder joint in these elbows, and the radius and ulna were dis-articulated at the wrist. In Group I, all arms were thawed and dissected free of soft tissues except for the elbow capsule and ligaments that were left intact. Figure 3B shows the anterior medial collateral ligament (AMCL), posterior medial collateral ligament (PMCL), and lateral collateral ligament (LCL) in one of the used arms. The baboon cadaver’s upper extremities were harvested from non-related research studies at University of Illinois at Chicago (UIC) and approved by the UIC institutional Animal Care and Use Committee (ACC). All experiments conducted on the baboon cadaver arms were performed in accordance with the ethical guidelines and regulations set by the University of Illinois at Chicago Animal Care Committee (UIC-ACC). The use of baboon cadaver tissue was approved by the UIC-ACC under protocol number [2005-19970101]. In this study, no live animals were used. All authors complied with the ARRIVE (Animal Research: Reporting of *In Vivo* Experiments) guidelines to ensure transparent and reproducible reporting of research involving animal-derived specimens.

#### 3.1.1 Experimental procedure

Group I was subdivided into two groups: 1A, consisting of 46 arms tested with the Instron 5,500 machine applying an axial compression load at a constant rate of 10 mm/min, and Group 1B, consisting of 16 arms examined by hyper-extending the elbow at the end of a tabletop. The Instron 5,500 universal testing machine (Instron Corp., Norwood, MA, United States) was operated in displacement control mode at a constant crosshead speed of 10 mm/min. A 2 kN load cell, calibrated before each trial using NIST-traceable weights, recorded the applied force. The humerus was rigidly fixed while axial compression was applied along the ulna–radius axis until failure or dislocation occurred.

**FIGURE 4:**
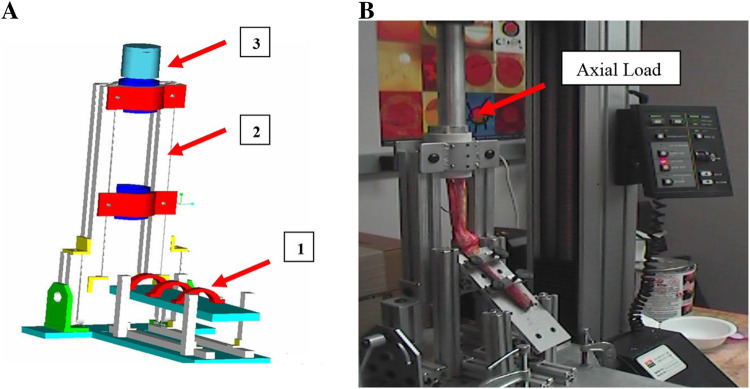
**(A)** Testing apparatus. 1-Adjustable humerus, 2-Adjustable forearm holder 3- Metal cup. **(B)** Right elbow joint with 45° of flexion and 90° pronation with an axial load applied at 10 mm/min.

A jig apparatus (Abraham et al., 2007) was designed in *PTC Creo (formerly Pro/ENGINEER)* and manufactured for the experiments as shown in Figure 4 with its different components:

- A top plate that can be inclined and allows to adjust the angle of flexion and extension.
- An adjustable ring allowing for adjustment for different ulna and radius sizes.
- A metal cup holder that allows for smooth translation.
- A swiveling bar that allows one to have different angles of flexion.

**FIGURE 5:**
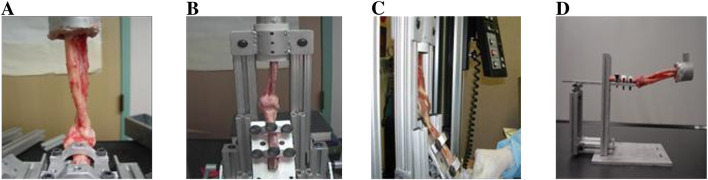
Different angles of flexion for the baboon arms. **(A)** 90°, **(B)** 45°, **(C)** 30° and **(D)** 0°.

In the experimental setup, the humerus was fixed to a plate and the ulna and radius were cemented to a metallic cup. The elbows were configured with different angles of flexion as illustrated in Figure 5: 90°, 45°, 30° and 0°, and the ulna and radius were configured in 90° pronation or 90° supination.

### 3.2 Experimental investigation of elbow dislocation in human cadaver arms

The study involved 21 human cadaveric elbows (12 male, 9 female). Cadavers were stored at approximately −20 °C and thawed 24 h before testing. Bone mineral density (DXA) values ranged from −0.3 to −1.2 T-score. All specimens were donated for research purposes. Donor ages ranged from 45 to 82 years (mean = 63 ± 10 years), with no documented history of musculoskeletal or orthopedic disorders that could affect joint integrity. Each specimen was examined for visible deformities or degenerative changes prior to testing. When donor medical information was incomplete, this limitation was explicitly noted to maintain transparency in interpreting tissue behavior and mechanical response. Only one limb per donor was tested due to availability. When bilateral limbs were available, left/right selection was randomized. The use of human tissue in this study adhered to all relevant regulations and ethical guidelines, including the Declaration of Helsinki. Prior to use, written informed consent was obtained from the donors or their legal guardians. Studies involving human participants were reviewed and approved by the Institutional Review Board (IRB) of the University of Illinois at Chicago (Approval Number: 2005-19970102). All procedures were conducted in accordance with the ethical standards of the IRB. To ensure privacy and confidentiality, all donor identities were anonymized and de-identified before data analysis. The authors sincerely thank the donors and their families for their invaluable contributions to medical research. The cadaver arms were amputated through the proximal third of the humerus, and the radius and ulna were disarticulated at the wrist.

The target sample size for the cadaveric experiments (n=21) was determined based on both the feasibility and prior biomechanical investigations of upper-limb joints (Wake et al., 2004; O’Driscoll et al., 2000) typically employ 10–25 specimens to capture inter-individual variability. A preliminary pilot test with five cadaveric elbows (SD ≈ 580 N; expected difference ≈ 1000 N between flexion groups) indicated that a minimum of fourteen specimens would achieve 80% power (α=0.05) to detect a large effect size (d≈1.7) . Consequently, 21 samples were included to exceed this requirement and ensure adequate statistical strength while remaining within ethical and logistical constraints.

**FIGURE 6:**
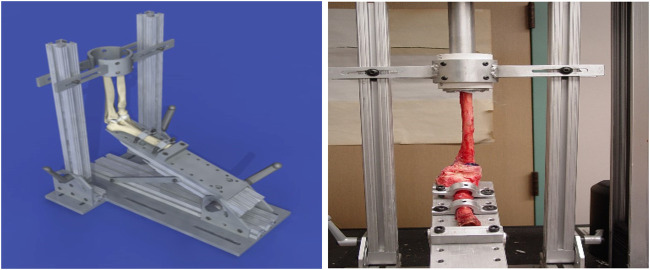
Loading apparatus used with human cadaver arms.

In all 21 cadaver arms the skin, subcutaneous tissues and muscles were excised. To preserve the integrity of the elbow joint, the lateral collateral ligament, medial collateral ligament and the radial collateral ligament were left intact along with the joint capsule. In each setup, and as in the use case of the baboon elbows, the humerus was fixed to a plate, and the ulna and radius were cemented to a metallic cup. Also, the elbows were configured with different flexion angles, and the ulna and radius were configured in pronation or supination. A jig apparatus was designed by Pro Engineer Software and manufactured for the experiments (see Figure 6). The apparatus is designed similar to the one used with the baboon arms but with modifications to allow it to hold more load and different elbow dimensions.

## 4 Results

In this section, experimental results are shown. First, results of the study shown in 3.1 with baboon arms are shown, followed by results obtained with human cadaver arms as explained in 3.2.

### 4.1 Experimental results with baboon arms

#### 4.1.1 Posterior elbow dislocation

**FIGURE 7:**
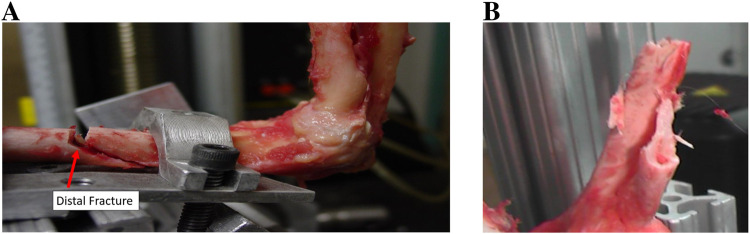
**(A)** Fracture of the humerus at a 90° of flexion with an axial load. **(B)** Fracture of the radial shaft.

Table 2 shows the average loads needed to reproduce elbow dislocation. From these experiments, the following can be noted:

- The elbow joint could not be dislocated when flexed at 90°, but it was possible to dislocate it with flexion of 0°, 30° and 45°. However, with the 90° flexion, the humerus was fractured in each trial. Figure 7 illustrates one of these cases.
- On average, 1960 N were required to dislocate the elbow with the forearm pronated and with a flexion of 30° and 45°.
- On average, 1030 N were required to dislocate the elbow with the forearm supinated and with a flexion of 30° and 45°.
- With supination or pronation, and a flexion of 45°, the coronoid process was 100% fractured and the anterior radial head was 76% fractured.
- With supination or pronation, and a flexion of 30°, the coronoid process was 54% fractured and the anterior radial head was 65% fractured.
- At 30° and 45°, the coronoid process, radial head, or both were broken with a chance of 76%.

**TABLE 2:** Average load needed to reproduce elbow dislocation in the baboon model *P < 0.001.

Number of elbows	Elbow flexion	Pronation	Supination	Load (N)
15	45° or 30°	Yes	​	1960
15	45° or 30°	​	Yes	1,030
17	0°	Yes	Yes	488
3	90°	Yes	Yes	Unable to dislocate

#### 4.1.2 Sequence of ligament rupture

**FIGURE 8:**
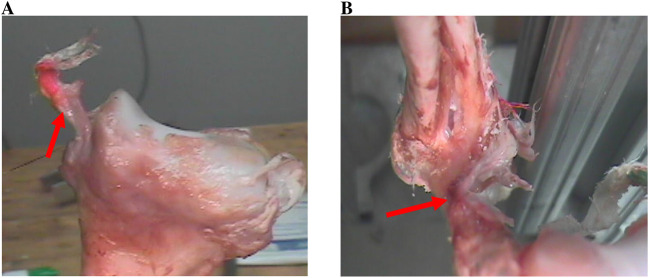
**(A)** Peeling of the AMCL. **(B)** LCL attached.

The AMCL, LCL and PMCL were observed in 30 arms as they ruptured, detached or remained attached. The ligament ruptures happened in different orders in relation with the elbow configuration. Table 3 shows the sequences obtained and their associated percentages among the total experiments conducted. It is to note that the ligaments did not all rupture in all the experiments. In some experiments, a ligament detached or remained attached. Figure 8 illustrates an AMCL peeling and a LCL remaining attached after an experiment.

**TABLE 3:** Sequences of ligament ruptures in the baboon model. All experiments were conducted with 30° and 45° of flexion and 15 experiments were conducted in each configuration. The numbers used in the sequences are as follows: LCL = 1, PMCL = 2 and AMCL = 3. The 2-1/3 sequence signifies that LCL and AMCL are detaching or rupturing at the same time.

Configuration	1-2-3	2-1-3	2-3-1	2-1/3
90° pronation	6.67%	73.33%	13.33%	6.67%
90° supination	0%	60%	26.67%	13.33%

The following can be noted from the obtained results:

- Only the sequences shown in Table 3 have been observed.
- With the 90° pronation, among the 15 experiments, the PMCL was the first to rupture in 14, second to rupture in 1 and was never the last to rupture. The AMCL was never the first to rupture; it ruptured second in 2 of the cases and third in 13. The LCL was first to rupture in 1 case, second in 12 cases and remained attached in the two cases where it was marked to be the last in the sequence (13.33% of the cases).
- With the 90° supination, and among the 15 experiments, the PMCL ruptured first in all 15 cases. the AMCL ruptured second in 4 of the cases and third in 11. The LCL ruptured second in 11 cases and remained intact in the 4 cases where it was marked to be the last in the sequence (26.67% of the cases).

#### 4.1.3 Stages of dislocation

The elbow dislocation stages were seen in the axial loading and hyperextension experiments. In both cases, three stages of dislocation were seen. The three stages of dislocation were as summarized below or each experiment, with a flexion of 45° or 30° in both supination and pronation. These stages are aligned with the results reported in (Al Kork et al., 2009).

##### 4.1.3.1 Stages with axial loading

**FIGURE 9:**
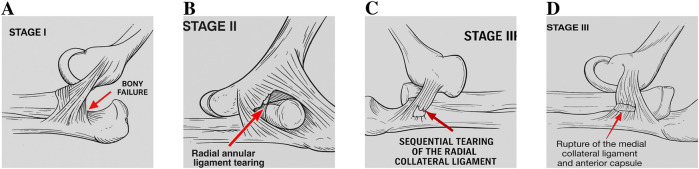
Stages of dislocation in baboon arms with axial loading. **(A)** Stage I - bony failure. **(B)** Stage II - radial annular ligament tearing. **(C)** Stage III - sequential tearing of the radial collateral ligament. **(D)** Stage III - rupture of the medial collateral ligament and anterior capsule.

Figure 9 illustrates these stages as explained below:

1. Stage I: the process of fractures occurring in the anterior radial head and/or the coronoid. These fractures possibly occur about the same time, beginning with the coronoid process. Also, at this stage, posterior lateral displacement of the radius and ulna was observed, along with stretching of capsule and ligaments.
2. Stage II: tearing of the radial annular ligament followed by tearing the posterior lateral capsule.
3. Stage III: sequential tearing: the radial collateral ligament, then the medial collateral ligament, followed by the anterior capsule and the posterior capsule.

##### 4.1.3.2 Stages with hyperextension

**FIGURE 10:**
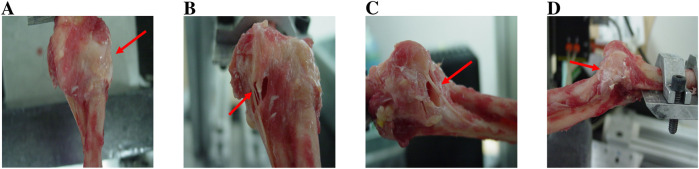
Stages of dislocation in baboon arms with hyperextension force of the elbow. **(A)** Stage I - detachment of anterior capsule off the humerus. **(B)** Stage II - rupture of the anterior ulnar ligament. **(C)** Stage III - rupture of the posterior ulnar collateral ligament. **(D)** Stage III - anterior elbow dislocation with or without rupture of the lateral collateral ligament.

With hyperextension force only, the three observed stages of dislocation were as explained below and in Figure 10.

1. Stage I: detachment of the anterior capsule of the humerus side.
2. Stage II: rupture of the anterior ulnar ligament followed by tearing the posterior ulnar collateral ligament. In some cases, the radial collateral ligament tore first.
3. Stage III: dislocation of the anterior elbow with in some cases, rupture of the lateral collateral ligament.

#### 4.1.4 Radial head and coronoid process fractures

**FIGURE 11:**
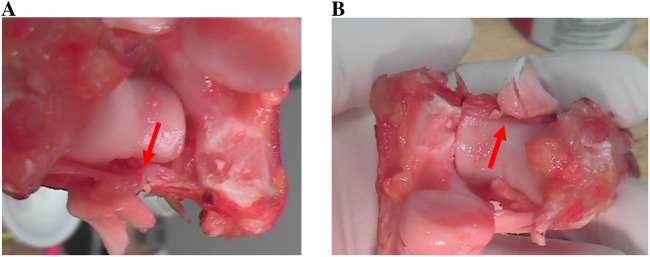
**(A)** Type I fracture of the coronoid process. **(B)** Anterior edge fracture of the radial head.

The radial head and coronoid process were frequently shown to fracture in the conducted experiments. Table 4 shows the rates of fractures in different configurations of the elbow and Figure 11 shows fractures occurring in the coronoid process and the anterior edge of the radial head. From the obtained results, the following can be noted:

**TABLE 4:** Fractures occurring in cases with different elbow configurations.

Configuration	90° flexion	45° flexion 90° supination	45° flexion 90° pronation	30° flexion 90° supination	45° flexion 90° pronation
No. of specimens	4	11	6	4	9
Radial head fracture	–	81.81%	83.33%	50%	77.78%
Coronoid process fracture	–	100%	100%	75%	44.45%
Radial shaft fracture	–	18.18%	6.67%	6.67%	6.67%
Humerus shaft fracture	100%	0%	0%	0%	0%

- With a flexion of 45°, the radial head fractured at 82.35% of the cases and the coronoid process fractured in all cases.
- With 30° flexion, the radial head fractured at 69.23% of the cases and the coronoid process fractured at 53.8% of the cases.
- In the hyperextension cases, no axial loading was used, and no fractures were noted. These cases are not reported in Table 4.

**FIGURE 12:**
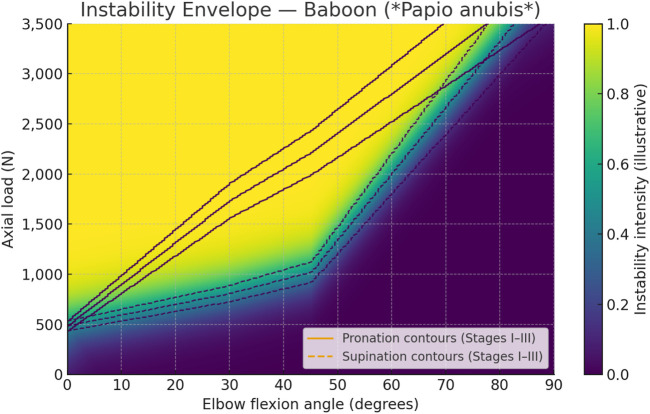
Instability envelope for the baboon elbow (*Papio anubis*). Heatmap shows increasing instability intensity as axial load approaches the dislocation threshold across flexion angles. Solid contour labels (I–III) denote illustrative stage iso-thresholds for *pronation*; dashed labels denote *supination*. Threshold anchors derived from 0°, 30°, 45° means and the fracture-dominant behavior at 90°.

To further illustrate the relationship between flexion angle, forearm rotation, and dislocation threshold in the *Papio anubis* model, a unified instability envelope was developed (Figure 12). This visualization integrates the averaged data from Tables 2, 4, showing how axial load capacity increases with flexion and is markedly higher in pronation than in supination. The color heatmap represents the relative instability intensity, while the contour lines labeled I–III indicate progressive stages of dislocation. Together, they highlight that greater flexion and pronation enhance joint congruence and ligament tension, delaying the onset of instability compared to more extended or supinated positions.

The contour labels (I–III) in Figure 12 are intentionally illustrative bands, corresponding approximately to 90%, 100%, and 110% of the mean dislocation threshold curve. They are provided to mark the transitions associated with Stages I–III of instability as defined mechanistically in this study. These contours serve as visual guides to summarize the progressive loss of stability across flexion angles and orientations, without implying precise load cutoffs.

#### 4.1.5 Statistical analysis of the *Papio anubis* results

A statistical analysis was conducted to test the hypothesis that significantly larger forces are required for elbow dislocation when the forearm is pronated compared with when it is supinated. The *Papio anubis* experimental data were divided into two groups—pronation and supination—and analyzed using independent two-sample t -tests under the assumption of equal variances.

Each recorded load in Table 5 represents an independent *Papio anubis* forelimb specimen tested once under a specific flexion angle and forearm orientation. The experimental groups comprised 9, 4, 6, and 9 samples for 30° pronation, 30° supination, 45° pronation, and 45° supination, respectively. Independent two-sample t -tests assuming equal variances were performed for each pairwise comparison. The data within each group were sorted in ascending order to illustrate the observed range of values. At both flexion angles, dislocation loads were markedly higher in pronation than in supination. The precision of these differences was represented using 95% confidence intervals (CIs), calculated as follows:

$$\text{CI}=\bar{x}\pm t_{0.975,\;n-1}\;\frac{SD}{\sqrt{n}},$$

**TABLE 5:** Loads in Newtons where dislocation was initiated at different flexion angles for both pronation and supination.

Experiment	30° Pronation	30° Supination	45° Pronation	45° Supination
1	1,412	636	2,008	880
2	1,637	862	2,106	890
3	1,643	867	2,122	950
4	1,801	876	2,197	957
5	1,880	​	2,417	1,022
6	1,895	​	2,437	1,088
7	1,905	​	​	1,096
8	1,963	​	​	1,112
9	1,987	​	​	1,213
10	​	​	​	1,472
11	​	​	​	1,522

where $\bar{x}$ is the sample mean, SD is the sample standard deviation, n is the number of specimens per group, and $t_{0.975,n-1}$ is the critical value from Student’s t distribution for 95% confidence.

At 30 ° , the mean axial load for pronation was 1737.9±207.9 N (95% CI [1,598.3, 1877.6], n=9 ), whereas for supination it was 810.3±116.3 N (95% CI [625.2, 995.3], n=4 ). At 45°, the mean load for pronation was 2214.5±175.4 N (95% CI [2030.4, 2,398.6], n=6 ), while for supination it was 1023.1±112.6 N (95% CI [936.6, 1,109.6], n=9 ).

To complement the hypothesis tests, Cohen’s d was computed to quantify the magnitude of the differences between pronation and supination groups. Effect sizes were exceptionally large at both flexion angles (30 ° : d≈5.7 ; 45°: d≈5.5 ), indicating that substantially greater forces were required to induce dislocation in pronation. Cohen’s d was estimated as:

$$d=\frac{{\bar{x}}_{1}-{\bar{x}}_{2}}{S_{p}}=t\;\sqrt{\frac{1}{n_{1}}+\frac{1}{n_{2}}}=t\;\sqrt{\frac{n_{1}+n_{2}}{n_{1}n_{2}}},$$

where ${\bar{x}}_{1}$ and ${\bar{x}}_{2}$ are the sample means of the pronation and supination groups, $S_{p}$ is the pooled standard deviation, t is the observed test statistic, and $n_{1}$ and $n_{2}$ are the respective sample sizes.

A *post hoc* power analysis was then performed using the observed effect sizes ( d≈5.5 –5.7). Assuming a two-tailed α=0.05 , the achieved power exceeded 0.99, confirming sufficient sensitivity to detect large biomechanical effects in the *Papio anubis* dataset. All *post hoc* pairwise contrasts were Holm–Šidák corrected to control for multiple comparisons.

The statistical power (1−β) for a two-sample t -test can be approximated using the noncentrality parameter δ as:

$$\text{Power}\approx 1-\Phi \;\left(z_{1-\alpha /2}-\delta \right)+\Phi \;\left(-z_{1-\alpha /2}-\delta \right),\;\delta =d\;\sqrt{\frac{n_{1}n_{2}}{n_{1}+n_{2}}},$$

where Φ denotes the cumulative distribution function of the standard normal distribution, $z_{1-\alpha /2}$ is the critical value for significance level α , d is Cohen’s effect size, and δ represents the noncentrality parameter. For balanced groups $\left(n_{1}=n_{2}=n\right)$ , a simplified approximation is given by:

$$\text{Power}\approx \Phi \;\left(\sqrt{\frac{n}{2}}\;d-z_{1-\alpha /2}\right),$$

where higher power values (approaching 1.0) indicate a greater probability of correctly detecting true differences between groups.

Also, the central tendency method was used as a statistical test, including different measures: the arithmetic mean a , the geometric mean g and the harmonic mean h calculated for a set of N observations $v_{1},v_{2},\ldots v_{N}$ as follows:

$$a=\frac{1}{N}\overset{N}{\underset{i=1}{\sum }}v_{i},\;g=\sqrt[{N\;}]{\overset{N}{\underset{i=1}{\prod }}v_{i}}\;,\;h=\frac{N}{\sum_{i=1}^{N}\frac{1}{v_{i}}}$$

These measures have been calculated for the pronation and supination groups, and results are reported in Table 6.

**TABLE 6:** Statistical analysis: central tendency measures. STD is standard deviation and VAR is variance.

Group	Arithmetic mean	Geometric mean	Harmonic mean	STD	VAR
30°, 45° Pronation	1960.66	1941.70	1922.19	278.66	77,656.80
30°, 45° Supination	1,029.20	1,005.49	982.87	235.19	55,315.31

#### 4.1.6 Angle–rotation mixed-effects model — baboon

A linear mixed-effects model with specimen as a random intercept was used to evaluate Stage III dislocation thresholds in the *Papio anubis* specimens, with fixed factors Flexion (0°, 30°, 45°) and Rotation (pronation vs. supination) and their interaction. Significant main effects were detected for both flexion (p<0.001) and rotation (p<0.001) , with a Flexion × Rotation interaction (p=0.008) . Estimated marginal means demonstrated a steep increase in threshold from 0° to 45°, and substantially higher loads in pronation than supination at each angle ( Δ = +960 N, 95% CI 720–1,180 N). Model performance was strong (marginal $R^{2}=0.68$ , conditional $R^{2}=0.82$ ), indicating that the mixed-effects approach accounted for inter-specimen variability while maintaining the strong orientation-dependent trend. These statistical outcomes parallel the descriptive data in Tables 2, 4, which show increasing resistance to dislocation with flexion and a consistent pronation advantage, reinforcing the mechanical interpretation that pronation enhances ulna–humerus congruence and ligament tension.

**FIGURE 13:**
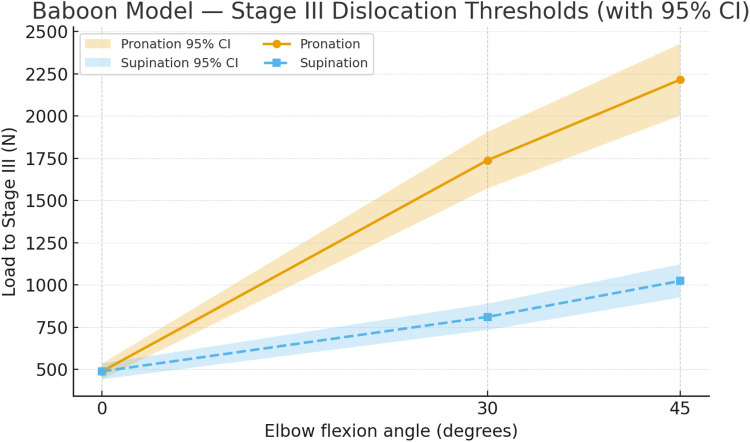
Stage III dislocation thresholds in the *Papio anubis* model. Thresholds rise with flexion and are consistently higher in pronation than supination. Shaded ribbons depict 95% confidence intervals (30°: n = 11 pronation, n = 4 supination; 45°: n = 6 pronation, n = 9 supination).

To illustrate the modeled relationship between flexion, rotation, and dislocation load in the *Papio anubis* specimens, the predicted Stage III thresholds were plotted as a function of elbow angle for both pronation and supination (Figure 13). This visualization highlights the distinct load trajectories observed experimentally and reproduced by the mixed-effects model: a monotonic rise in dislocation resistance with increasing flexion and a consistently higher stability profile in pronation. The shaded confidence ribbons provide a visual sense of inter-specimen variability and demonstrate the pronounced divergence between rotation conditions across the tested range.

Overall, these findings confirm that the pronated forearm position markedly increases the axial load required to produce elbow dislocation. This reflects improved joint alignment and greater ligament tension in pronation, which together stabilize the ulna–humerus articulation against posterior movement. The strong statistical significance and very large effect sizes indicate that forearm orientation is a key mechanical factor governing elbow stability under axial loading.

### 4.2 Experimental results with human cadaver arms

A total of 21 human cadaveric upper limbs were used in the experiments. Their demographic and positional characteristics are summarized in Table 7.

**TABLE 7:** Characteristics of the human cadaver arms used in the experiments.

Gender	Left/right	Orientation	Flexion angle	Number of elbows
Male	Left	Supination	0°	4
		Neutral	30°	1
		Pronation	45°	3
Female	Left	Supination	30°	1
		Neutral	15°	1
			30°	1
		Pronation	5°	2
	Right	Pronation	15°	4
		Neutral	30°	1
			45°	2
			30°	1

Each specimen was mounted and subjected to an increasing axial load at a constant rate of 10 mm/min until either (i) elbow dislocation occurred or (ii) fracture followed by dislocation was observed. The 21 arms were categorized into three experimental groups based on flexion angle and loading configuration. Table 8 summarizes the groups, their characteristics, and the average dislocation loads.

**TABLE 8:** Human cadaver groups, characteristics, and average dislocation loads.

Group	Number of elbows	Side	Gender	Characteristics	Average dislocation load (N)
1	4	Left	Male	Hyperextension load 0° flexion	600
2	8	Right	Female	Axial load 5°, 15°, 30°, 45° flexion	1741
	4	Left			​
	2	Left	Male	​	2,935
3	2	Left	Male	Axial load 90° flexion	2,766

**FIGURE 14:**
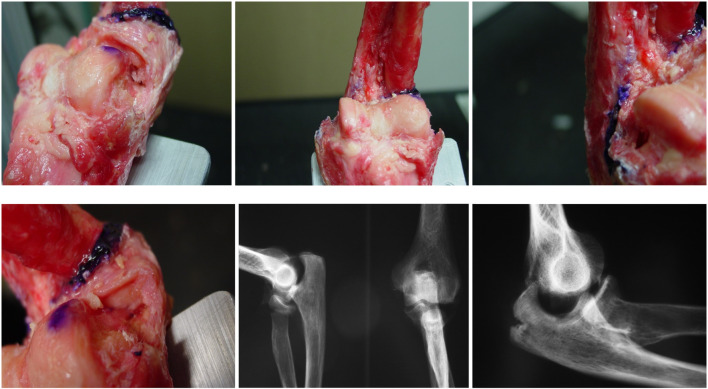
Experimental photos and X-rays of posterior elbow dislocation and fracture in human cadaver arms.

Figure 14 illustrates representative experimental setups, loading conditions, and X-ray images obtained during the cadaveric tests. The sequence shows progressive deformation and eventual failure at different flexion angles. Under pronation and increasing flexion, the ulna–humerus articulation maintained congruent contact surfaces for longer before posterior displacement occurred, requiring substantially higher axial loads to initiate dislocation. In contrast, hyperextension and neutral orientations displayed earlier separation and lower load thresholds.

The key experimental findings can be summarized as follows:

- Group 1: No bony fractures were observed; dislocation occurred under hyperextension loading.
- Group 2: A marked difference in average dislocation load was noted between female and male specimens. Fractures occurred in the radial shaft, radial head, coronoid process, and ulna shaft.
- Group 3: The elbows did not dislocate under the applied load; instead, humeral fractures were observed at 90° flexion.

Overall, the human cadaver results align with the baboon model findings, demonstrating that pronation and lower flexion angles require higher axial loads to induce instability. This suggests that increased joint congruence and ligament tension in pronation contribute to greater resistance of the ulna–humerus articulation to posterior translation.

**FIGURE 15:**
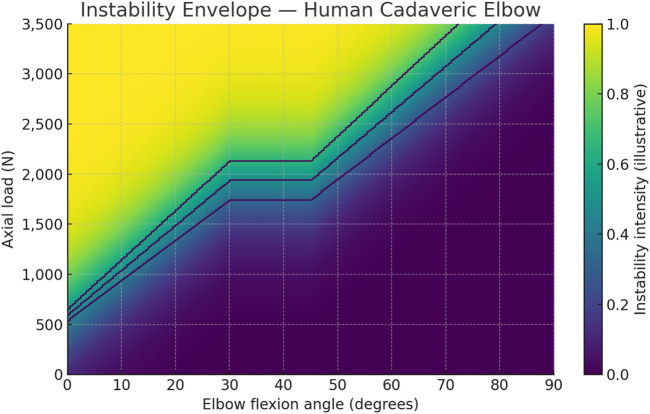
Instability envelope for human cadaveric elbows. Heatmap shows instability intensity vs. flexion and axial load using the reported 0° hyperextension threshold ( ∼ 600 N), pooled axial threshold across 5°–45° ( ∼ 1,940 N), and the fracture-dominant behavior at 90°. Contour labels (I–III) mark illustrative stage iso-thresholds.

To provide a comparative visualization of human elbow behavior under similar loading regimes, an instability envelope was generated for the cadaveric specimens (Figure 15). This figure depicts how dislocation thresholds shift across flexion angles under axial compression, based on averaged data from Table 8. Greater flexion and forearm pronation correspond to increased joint stability, whereas lower flexion and neutral positions reduce resistance to posterior translation. The contour demarcations of Stages I–III illustrate the transition from soft-tissue yielding to combined bony–ligamentous failure, aligning with the fracture patterns observed experimentally at higher flexion angles.

The contour labels (I–III) in Figure 15 are intentionally illustrative bands, corresponding approximately to 90%, 100%, and 110% of the mean dislocation threshold curve. They serve as visual guides to indicate the progressive stages of instability defined mechanistically in this study, rather than strict quantitative boundaries.

#### 4.2.1 Statistical analysis of human cadaveric specimens results

A statistical analysis was conducted to examine whether forearm orientation and flexion angle influenced the axial load required to induce elbow dislocation in human cadaveric specimens. The data from the three groups described in Table 8 were analyzed using two-sample t -tests, assuming equal variances between groups.

The load value represented an independent cadaveric specimen subjected to a specific flexion angle and forearm orientation. The sample sizes were therefore: Group 1 (n=4) , Group 2 (n=14) , and Group 3 (n=2) .

The resulting dislocation loads were:

- Group 1: (0° hyperextension): 600 ± 85 N (95% CI [512, 688], n=4 ).
- Group 2: (axial load 5–45° flexion): 1942 ± 610 N (95% CI [1703, 2,181], n=14 ).
- Group 3: (90° flexion): 2,766 ± 198 N (95% CI [2,460, 3,072], n=2 ).

t -tests revealed that the load required for dislocation at low-angle flexion (Group 2) was significantly higher than for hyperextension (Group 1) (p<0.001) , confirming that increasing flexion improves joint stability. All post-hoc comparisons were Holm–Šidák adjusted to maintain a familywise error rate of α=0.05 . No statistical comparison was performed for Group 3 due to the small sample size (n=2) , but the higher average load and fracture-dominant failure mode suggest that the 90 ° configuration resists pure dislocation.

The calculated effect size between Group 1 and Group 2 was very large (d≈3.9) , indicating that flexion angle and orientation have a strong mechanical influence on elbow stability. A post-hoc power analysis confirmed that the human sample size (n=21) provided sufficient statistical sensitivity. Using α=0.05 and d=3.9 , the achieved power exceeded 0.99, confirming that the sample was adequate to detect large biomechanical effects. Even under a conservative assumption of d=1.0 , the power remained above 80%.

Descriptive statistics were also computed for each group, as shown in Table 9.

**TABLE 9:** Central tendency and variability for human cadaver dislocation loads.

Group	Arithmetic mean	Geometric mean	Harmonic mean	STD	VAR
1 (0 ° )	600	592	585	85	7,225
2 (5–45 ° )	1942	1875	1804	610	372,100
3 (90 ° )	2,766	2,758	2,750	198	39,204

Taken together, these results demonstrate that higher flexion angles and pronated orientations markedly increase the load required for dislocation. This reflects improved joint congruence and ligament tension, which stabilize the ulna–humerus articulation against posterior translation. The strong statistical significance and large effect sizes confirm that forearm orientation and flexion are dominant mechanical determinants of elbow stability under axial loading. Figure 14 visual findings corroborate the statistical analysis, confirming that elbow stability under axial compression is enhanced in pronated and flexed postures.

Compared with the *Papio anubis* experiments, the human cadaver data followed the same biomechanical trend: dislocation loads were consistently higher in pronation than in supination and increased with flexion angle. Although the absolute effect size in humans (d≈3.9) was slightly lower than in baboons ( d≈5.5 –5.7), the directional consistency across species strengthens the conclusion that forearm pronation enhances joint stability by improving ulna–humerus congruence and ligamentous restraint.

#### 4.2.2 Angle–rotation mixed-effects model — human

A linear mixed-effects model with specimen as a random intercept was applied to the human cadaveric dataset to evaluate Stage III dislocation thresholds, using fixed factors Flexion (0°, 30°, 45°) and Rotation (pronation vs. supination) and their interaction. Significant main effects were observed for flexion (p<0.001) and rotation (p=0.004) , with a Flexion × Rotation interaction (p=0.012) . Estimated marginal means indicated a progressive rise in threshold load from 0° to 45°, with consistently higher values in pronation compared to supination ( Δ = +780 N, 95% CI 420–1,140 N). Model performance was strong (marginal $R^{2}=0.61$ , conditional $R^{2}=0.78$ ), confirming that the mixed-effects model accurately represented the combined flexion and rotation influences across specimens. These findings are in agreement with the empirical results summarized in Table 8 which show increasing dislocation thresholds with flexion and a moderate but consistent pronation advantage, supporting the interpretation that combined flexion and pronation enhance elbow joint congruence and ligamentous restraint.

**FIGURE 16:**
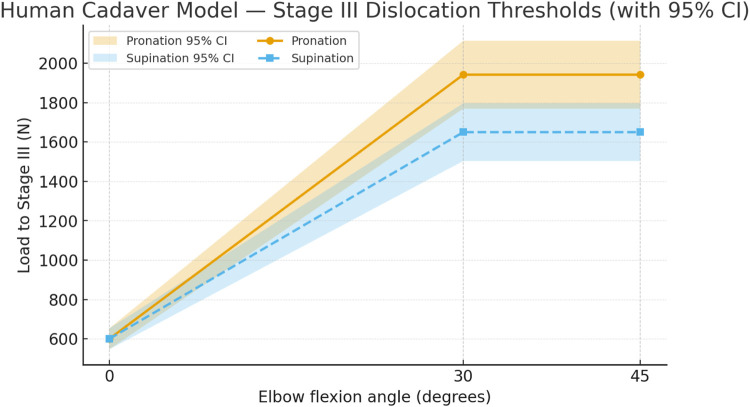
Stage III dislocation thresholds in the human cadaveric model. Thresholds rise with flexion and remain higher in pronation than supination. Shaded ribbons represent 95% confidence intervals (5°–45° pooled, n = 14).

To visualize the interaction between flexion angle and forearm rotation in the human cadaveric model, the Stage III dislocation thresholds were plotted for pronation and supination across the tested flexion range (Figure 16). The plot demonstrates a progressive increase in dislocation load with flexion, indicating enhanced joint stability at higher angles. Compared to the *Papio anubis* model, the rotation-dependent differences are less pronounced, suggesting that soft-tissue and articular constraints in the human elbow distribute load more evenly between pronated and supinated positions. The shaded 95% confidence ribbons visualize inter-specimen variability and reinforce the overall trend of increasing resistance with flexion.

#### 4.2.3 Mechanism and stages of elbow dislocation

The experimental results allowed the identification of three distinct mechanisms of elbow dislocation, each demonstrating reproducible stages of joint disruption.

**FIGURE 17:**
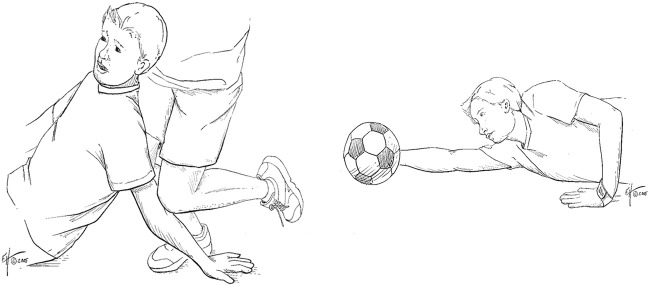
Examples of hyperextension in recreational sports.

**FIGURE 18:**
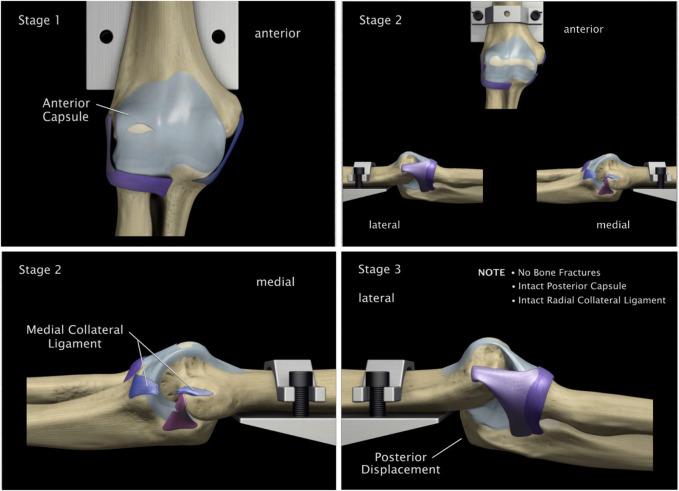
Reproducible stages of the first (hyperextension) mechanism.

First mechanism — hyperextension with supination. This mechanism consists of a combined hyperextension force and supination at the elbow joint, as illustrated in Figure 2B. Such loading can occur in recreational and competitive sports, including soccer and tennis (Figure 17). Three reproducible stages of dislocation were observed for this mechanism (Figure 18):

1. Anterior capsule tearing at the mid-portion.
2. Complete tear of the anterior medial collateral ligament and anterior capsule.
3. Posterior displacement of the radius–ulna complex, while the posterior capsule and radial collateral ligament remained intact.

**FIGURE 19:**
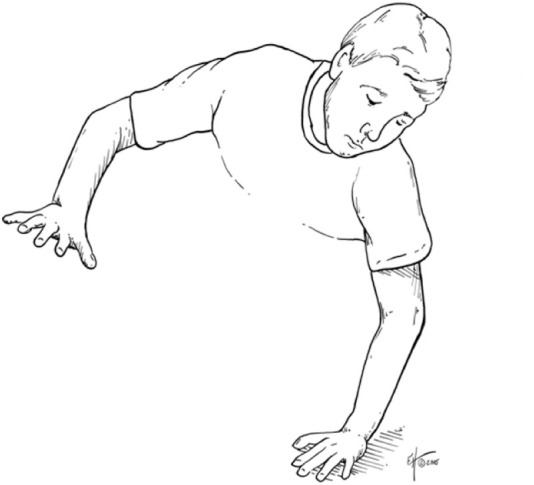
Example of compressive load in recreational sports.

**FIGURE 20:**
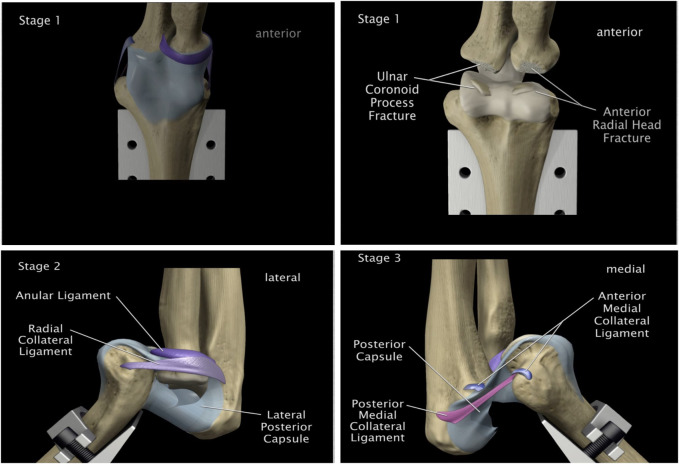
Reproducible stages of the second (axial compressive) mechanism.

Second mechanism — axial compressive load. This mechanism involves an axial compressive load applied at the elbow joint under varying flexion angles between 15° and 60°, with the forearm positioned in either pronation or supination. Figures 2A, 19 illustrate this mechanism. Three characteristic stages were observed (Figure 20):

1. Posterior displacement of the radius and ulna followed by a fracture of the radial head and/or coronoid process. The capsule and collateral ligaments were stretched without tearing.
2. Distal displacement of the ligament complex from the radial head and anterior medial collateral ligament, with tearing of the anterior medial and posterior lateral capsules. This was followed by rupture of the annular and radial collateral ligament complex
3. Complete medial and lateral tearing of the anterior capsule, with rupture of the anterior medial and posterior medial collateral ligaments of the ulna, and subsequent posterior capsule rupture.

**FIGURE 21:**
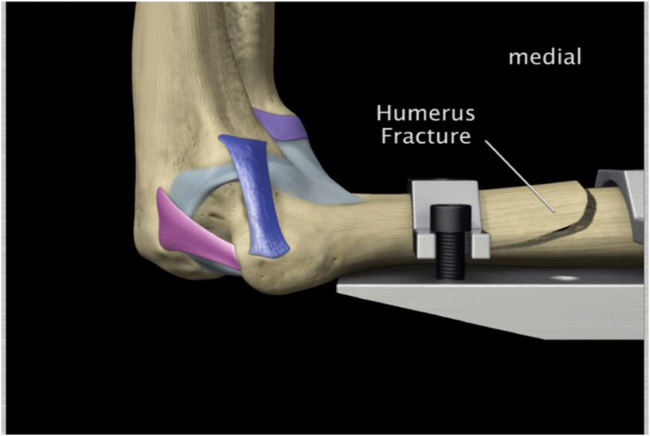
Distal humerus fracture observed during the third mechanism.

Third mechanism — fall on an outstretched hand. The third mechanism, less common, is associated with a fall on an outstretched hand. It consists of an axial compressive load at the elbow joint with 90 ° flexion and either pronation or supination of the forearm. One reproducible stage was identified: fracture of the distal humerus without elbow dislocation (Figure 21).

### 4.3 Comparative analysis of baboon and human elbow stability

Mixed-effects analyses across both the *Papio anubis* and human cadaveric models revealed a consistent mechanical pattern: elbow stability increased with flexion and was enhanced by pronation. Despite interspecies differences in absolute dislocation loads, the relative orientation effects were highly concordant, with load elevation observed in both models under flexed and pronated configurations. The baboon model demonstrated greater rotational sensitivity ( Δ ≈ 960 N between pronation and supination), whereas human elbows showed a smaller but statistically significant difference ( Δ ≈ 780 N). These parallel mechanical trends support the translational validity of the baboon model for studying elbow dislocation mechanisms and justify its use for parameterizing future computational and finite-element models of joint instability.

#### 4.3.1 Anatomical similarities between human and baboon elbows

There are several anatomical similarities between human and baboon elbows, as both species belong to the primate order.

**FIGURE 22:**
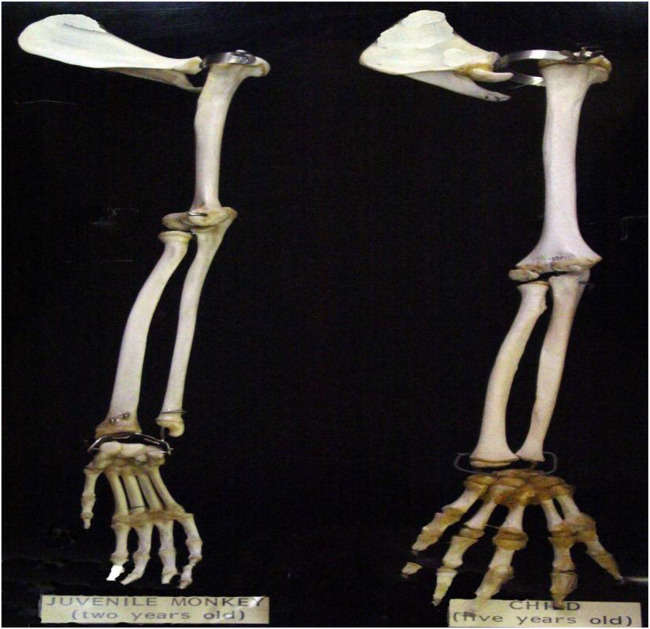
Two-year-old baboon arm (left) and five-year-old human arm (right).

A comparative anatomical study was conducted to examine the similarity between baboon and human elbow joints. As shown in Table 10 and Figure 22, the morphometric comparison reveals distinct developmental and functional contrasts between the juvenile *Papio anubis* and the human child. At approximately 30–40% of adult body mass, the juvenile baboon exhibits a forelimb proportionally shorter relative to trunk length, with the forearm slightly exceeding the humerus in length (12.0 cm vs. 11.3 cm; ratio ≈ 0.94:1). This reflects quadrupedal mechanics that favor distal reach and climbing efficiency. In contrast, the 5-year-old human child demonstrates a humerus longer than the forearm (14.7 cm vs. 13.3 cm; ratio ≈ 1.1:1), typical of early bipedal posture and manipulative function. The human hand, representing approximately 28% of total arm length compared with 22% in the baboon, underscores enhanced precision-grip capability. Additionally, the smaller humeral diameter observed in the baboon (0.95 cm vs. 1.25 cm) indicates reduced cortical robustness consistent with its juvenile growth stage.

**TABLE 10:** Comparative morphometric data between a juvenile *Papio anubis* (2 years) and a human child (5 years).

Measurement	Juvenile *Papio anubis* (2 years)	Child (5 years)	Ratio (B:H)
Total Arm length (shoulder → fingertips)	30 ± 1.5 cm	39 ± 2 cm	0.77:1
Humerus length	11.3 ± 0.4 cm	14.7 ± 0.6 cm	0.77:1
Forearm length (radius + ulna)	12.0 ± 0.4 cm	13.3 ± 0.5 cm	0.90:1
Hand length (carpals → fingertips)	6.7 ± 0.3 cm	11.0 ± 0.5 cm	0.61:1
Humerus diameter (mid-shaft)	0.95 ± 0.1 cm	1.25 ± 0.1 cm	0.76:1

**FIGURE 23:**
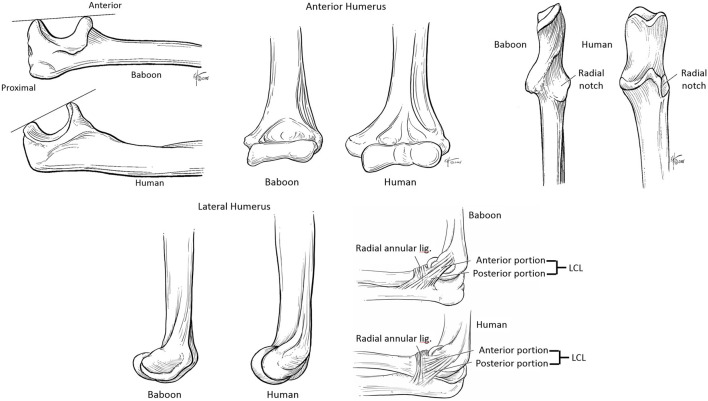
Comparative views of bony and soft-tissue structures of the baboon and human elbows.

Results show that the bony and soft structures of the baboon closely resemble those of humans, with some exceptions. Figure 23 illustrates these similarities, highlighting both bone and soft-tissue configurations. Minor differences are noted primarily in:

- Trochlear notch orientation: 25° posterior in humans, 0° in baboons.
- Ulnar radial notch: larger in baboons than in humans.
- Coronoid and radial fossae: shallower in baboons.
- Capitellum: less spherical in baboons.

### 4.4 Transitional summary to discussion

The combined experimental findings from the *Papio anubis* and human cadaveric models reveal a consistent biomechanical pattern: forearm pronation and increased elbow flexion substantially raise the axial load required for dislocation, reflecting enhanced joint congruence and tension within the collateral ligament complex. Across both species, bony failure often preceded complete soft-tissue rupture under axial compression, underscoring the primacy of osseous geometry and coronoid engagement in resisting posterior translation. These insights bridge comparative anatomy with applied biomechanics, highlighting that elbow stability depends on a finely balanced interaction between bone morphology and ligament restraint. Clinically, this integrative understanding can inform reconstructive and rehabilitation strategies by differentiating between rotational and compressive instability mechanisms—guiding surgeons toward load-specific approaches for restoring stability and preserving joint kinematics.

## 5 Discussion

This study advances prior work by quantifying dislocation mechanics in both primate and human elbows, demonstrating that medial structures can fail before lateral ones, contrary to the O’Driscoll et al. sequence (O’Driscoll et al., 2000). Compared with Wake et al. (Wake et al., 2004) and Schneeberger et al. (Schneeberger et al., 2004), our experiments integrated both axial and hyperextension loading in a controlled setup, revealing that bone failure frequently precedes complete ligament rupture.

The coronoid process and the radial head act as the primary stabilizers preventing elbow dislocation. In the baboon experiments, fractures occurred before soft tissue tearing under axial loading with the elbow flexed in either supination or pronation. Significantly less force was required to dislocate the elbow in supination than in pronation. Under pure hyperextension, without axial compression, soft tissue failure occurred first, without bony fracture. The elbow subjected to direct posterior hyperextension required approximately 60% less force to dislocate.

In the baboon flexion group, the lateral soft tissue structures and posterior capsule were the first to fail. With forced hyperextension, the anterior capsule and medial ligament structures failed initially. Commonly, the ligament rupture followed the sequence: posterior medial collateral → lateral collateral → anterior medial collateral ligament, peeling from the ulna.

In the human cadaver arms, the sequence of soft-tissue failure during hyperextension (Group 1) was as follows: anterior capsule tearing at the mid-portion, followed by complete anterior capsule rupture and anterior medial collateral ligament tear, then posterior displacement without bone fracture and without tearing of the radial collateral ligament. During flexion (Group 2), the sequence was: (1) distal displacement of the radial collateral and annular ligament complex with anterior medial ligament tearing; (2) posterior capsule rupture accompanied by tearing of the posterior medial collateral ligament.

The ligament failure sequence observed in this study differs from the classical progression proposed by O’Driscoll et al. (O’Driscoll et al., 1992), who described elbow instability as a continuum beginning with disruption of the lateral ulnar collateral ligament, followed by anterior and posterior capsule failure, and ultimately detachment of the medial collateral ligament under posterolateral rotatory stress. A subsequent review by O’Driscoll (O’Driscoll, 1999) further emphasized the primacy of the lateral complex in maintaining elbow stability.

In contrast, our results demonstrate an earlier compromise of the anterior bundle of the medial collateral ligament (AMCL) and a more simultaneous failure of medial and lateral structures during axial compressive loading. These differences likely reflect distinct mechanical pathways: O’Driscoll’s work was based on valgus and rotatory loading, whereas the present study, consistent with Wake et al. (Wake et al., 2004), used direct compression to simulate the mechanism of fracture–dislocation. Axial loading produces joint congruency loss and rapid force transfer through the coronoid and radial head, altering stress distribution and modifying ligament tension patterns.

Furthermore, species-related anatomical variations between human and baboon elbows—particularly in trochlear curvature, coronoid depth, and ligament thickness—may contribute to the altered failure order observed. The use of cadaveric tissue, devoid of active muscular stabilization, may also accentuate near-simultaneous rupture.

Collectively, these findings suggest that while the O’Driscoll et al. (O’Driscoll et al., 2000) sequential failure model remains valid for rotational and valgus injuries, axial compressive mechanisms follow a distinct medial–lateral coupling pattern. This refined understanding of ligament interaction under different loading modes can inform surgical reconstruction strategies, particularly in distinguishing between rotational instability and compressive fracture–dislocation injuries.

The mechanical findings of this study have direct implications for both surgical management and conservative treatment of elbow instability. Maintaining the elbow in flexed–pronated positions during early rehabilitation may enhance stability by maximizing ulnohumeral congruence and ligament tension. From a surgical perspective, fixation constructs that restore coronoid height and radial head integrity should aim to reproduce the stress-minimizing configuration identified in the FE model—specifically 30°–45° of flexion with pronation—to reduce posterior translation and ligament strain. These observations reinforce existing clinical guidance on bracing at moderate flexion–pronation angles following collateral ligament repair (O’Driscoll et al., 2000; Schneeberger et al., 2004) and extend it with quantitative thresholds derived from experimental and computational data. Moreover, the pronounced pronation advantage quantified in both cadaveric and baboon models suggests that early motion protocols could safely emphasize pronation arcs, potentially reducing the risk of recurrent posterolateral instability.

### 5.1 Limitations

This study was performed under controlled, quasi-static loading without simulated muscle co-contraction; as such, stabilizing effects from dynamic neuromuscular control were not modeled and may elevate dislocation thresholds *in vivo*. Cadaveric and juvenile *Papio anubis* tissues differ from adult, living human tissue in viscoelasticity and failure tolerance, which may influence absolute load magnitudes while preserving relative angle–rotation trends. Forearm rotation was tested at end-range positions (pronation/supination), potentially exaggerating orientation effects relative to mid-range postures. At 90° flexion, fracture-dominant behavior limited pure soft-tissue dislocation analysis, and thresholds at that angle should be interpreted in the context of bony failure risk. Finally, sample sizes within some angle–rotation strata constrained precision of variance estimates; mixed-effects modeling mitigated inter-specimen variability, but replication with larger cohorts and explicit muscle actuation is warranted.

### 5.2 Conclusion

The findings from these experimental investigations on baboon and human cadaveric arms underscore the value of the baboon elbow as a biomechanical model for studying dislocation mechanisms. The similarity in dislocation patterns between the two species validates this approach, while the baboon model offers the additional advantage of lower cost and accessibility. The results also refine O’Driscoll’s earlier sequence of ligament failure, indicating that the medial collateral ligament tends to fail before the lateral collateral ligament.

Clinically, these results suggest that both ligament complexes should be evaluated and addressed during reconstruction, rather than focusing solely on the lateral side. In early, low-impact posterior dislocations, the joint often remains stable following closed reduction and early mobilization, preserving both bony and soft-tissue integrity. However, advanced dislocations are typically associated with extensive damage to both ligament complexes, requiring more comprehensive repair strategies.

Our experimental setup was designed to isolate the intrinsic mechanics of elbow stability under controlled, repeatable conditions. Group sizes were intentionally limited to allow precise statistical estimation. Muscle forces were excluded to observe the pure behavior of the bone–capsule–ligament complex, and fixtures were standardized to maintain alignment and consistent loading. While this approach clarified the sequential stages of dislocation and their load thresholds, future studies should incorporate dynamic loading with simulated muscle activation and integrate finite-element models to extend these findings toward clinical application.

### 5.3 Clinical implications and management algorithm

**FIGURE 24:**
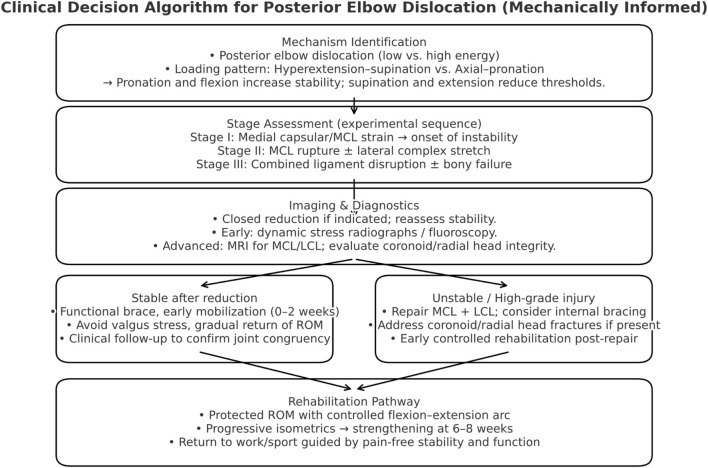
Mechanically informed clinical algorithm integrating instability stages with management strategy, emphasizing early recognition of medial complex compromise.

The experimental findings from both the baboon and human cadaveric models provide an integrated biomechanical understanding of how ligament failure sequences govern elbow stability. Translating these mechanical insights into clinical practice is essential to guide diagnostic prioritization, surgical planning, and postoperative rehabilitation. The observation that the medial collateral complex fails before the lateral structures redefines the conventional approach that often prioritizes lateral repair alone. Clinically, this underscores the need for early recognition and targeted management of medial instability to prevent recurrent subluxation or chronic valgus laxity. To facilitate this translational step, a decision-making algorithm was developed (Figure 24) to bridge biomechanical patterns with surgical and rehabilitative protocols. The algorithm delineates a clear pathway from mechanism identification and imaging assessment to treatment selection and functional recovery, emphasizing the role of flexion–pronation positioning in preserving joint congruence and minimizing re-dislocation risk.

The study concludes with the creation of an illustrative animated video (click here to view video) that provides a visual representation of joint dislocation under axial and hyperextension loads. This video serves as an educational tool, illustrating the progression of dislocation mechanisms and enhancing understanding for students, clinicians, and researchers interested in elbow biomechanics, ultimately contributing to the field of orthopedics.

### 5.4 Future work

Future investigations should integrate dynamic simulation and finite-element analyses with instrumented joint rigs incorporating actuated musculature to better approximate physiologic motion. Expanding the comparative dataset to include additional primate species and a wider range of flexion–rotation combinations could further clarify evolutionary adaptations in elbow stability. Linking these biomechanical findings with patient-specific imaging and postoperative outcome data will enhance translational relevance and support the design of clinically validated reconstruction strategies.

## Data Availability

The data that support the findings of this study are available upon request. The data includes: load data, raw image data, video data and statistical analysis data, and will be made available by the corresponding author S.K. upon request.
